# Supplementary figures and images for: Buffered EGFR signaling regulated by spitz-to-argos expression ratio is a critical factor for patterning the Drosophila eye
Source: PLoS Genet. 2023 Feb 2;19(2):e1010622. doi: 10.1371/journal.pgen.1010622 (PMC9928117; doi:10.1371/journal.pgen.1010622)

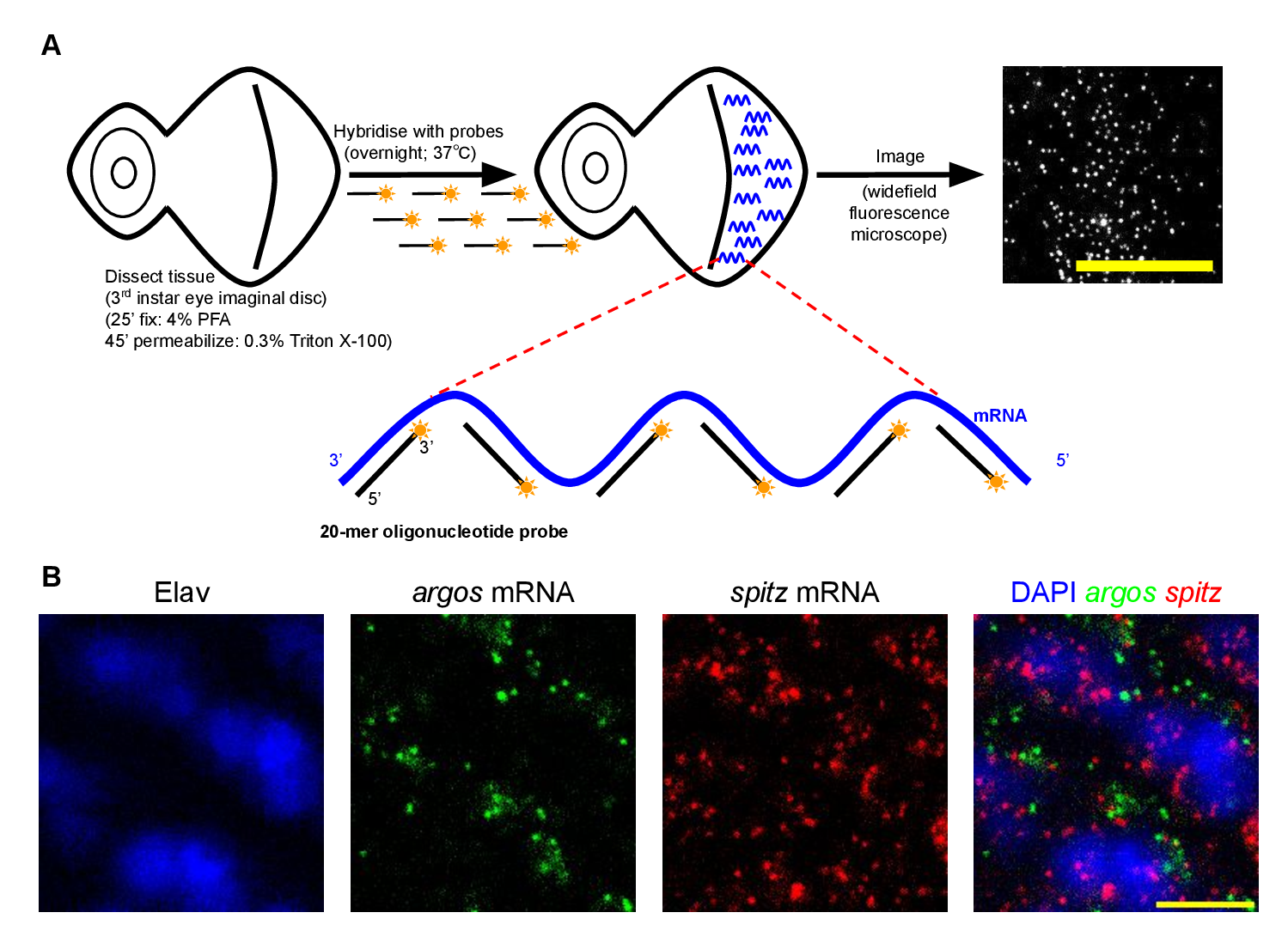

Supplement: S1 Fig — (A) Cartoon showing single molecule RNA FISH protocol. Tissues are fixed and permeabilized and hybridized overnight with complementary probes. Multiple 20 nt long oligos each carrying a 3’ fluorophore is used to decorate the mRNA of interest following previous protocols. Singly-labeled 20-mer probes bind along the mRNA length and appear as diffraction-limited spots when imaged. Scale bar is 5μm. (B) Zoomed images of a single z-slice showing single transcripts of spitz mRNA and argos mRNA. Scale bar is 5μm. The images within the figure panels are created by the authors. (TIFF) [file pgen.1010622.s001.tiff]

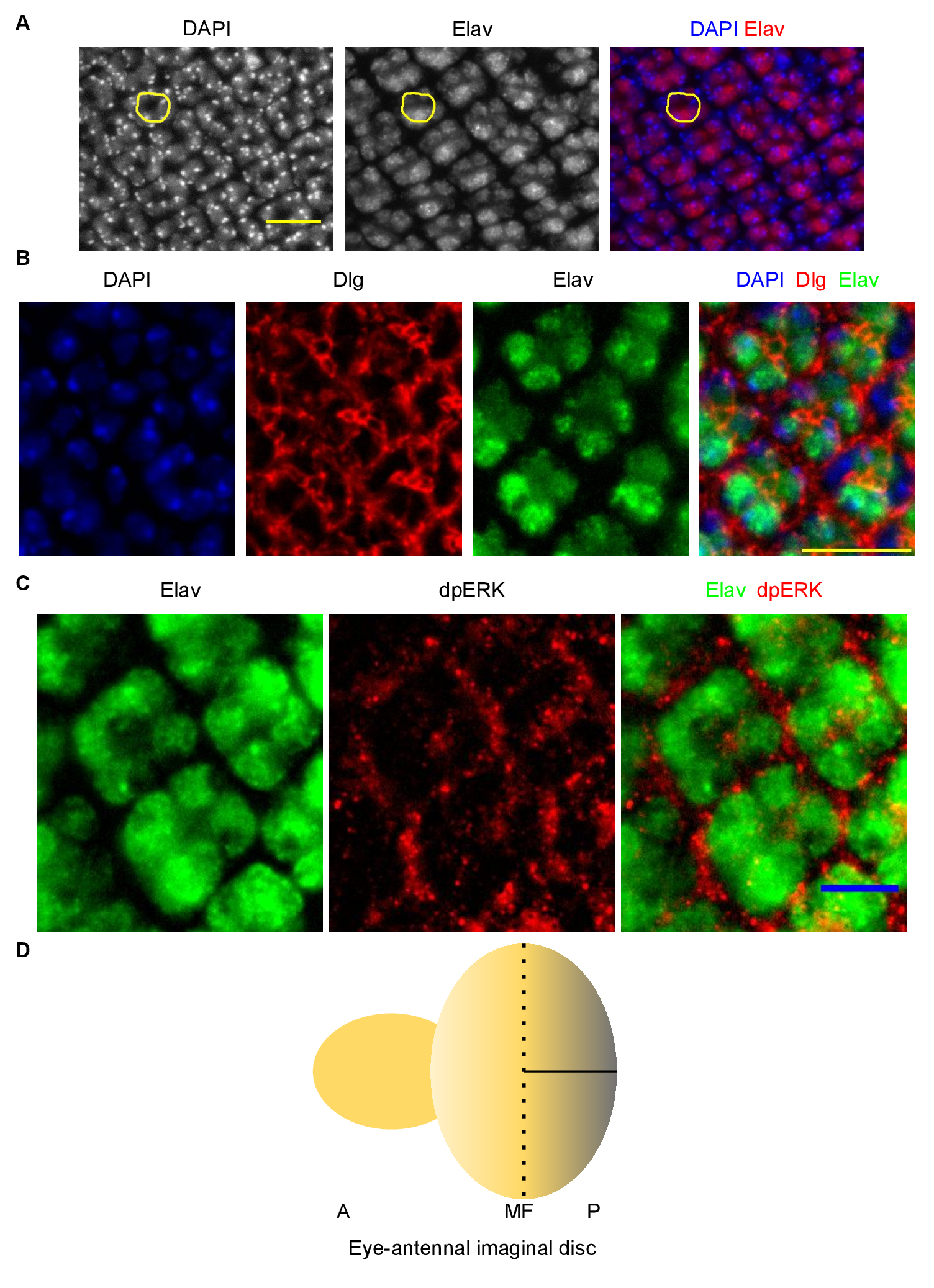

Supplement: S2 Fig — (A) mRNA was counted specifically in the photoreceptor using the rosette like arrangement of PR nuclei. The marked yellow region corresponds to one photoreceptor cluster marked in DAPI channel which also stains positive for a pan-neuronal marker, Elav. (B) CantonS 3rd instar larval eye disc stained with Dlg and Elav antibody. The nuclei around the clusters of Elav-positive neuronal photoreceptor (PR) cells, are the non-photoreceptor (non-PR) cells. The non-PR cells are also separated by Dlg which stains the membrane. (C) The Elav-positive cells and cells stained with dp-ERK are exclusive, again saying that EGFR signaling is activated in the neighbouring cells to the ligand source of PR cells. Scale bar is 10μm in (A) and 5μm in (B and C). (D) Cartoon representing the line along which spitz and argos mRNA counts were analysed (A: anterior end; MF: morphogenetic furrow; P: posterior end). The images within the figure panels are created by the authors. (TIFF) [file pgen.1010622.s002.tiff]

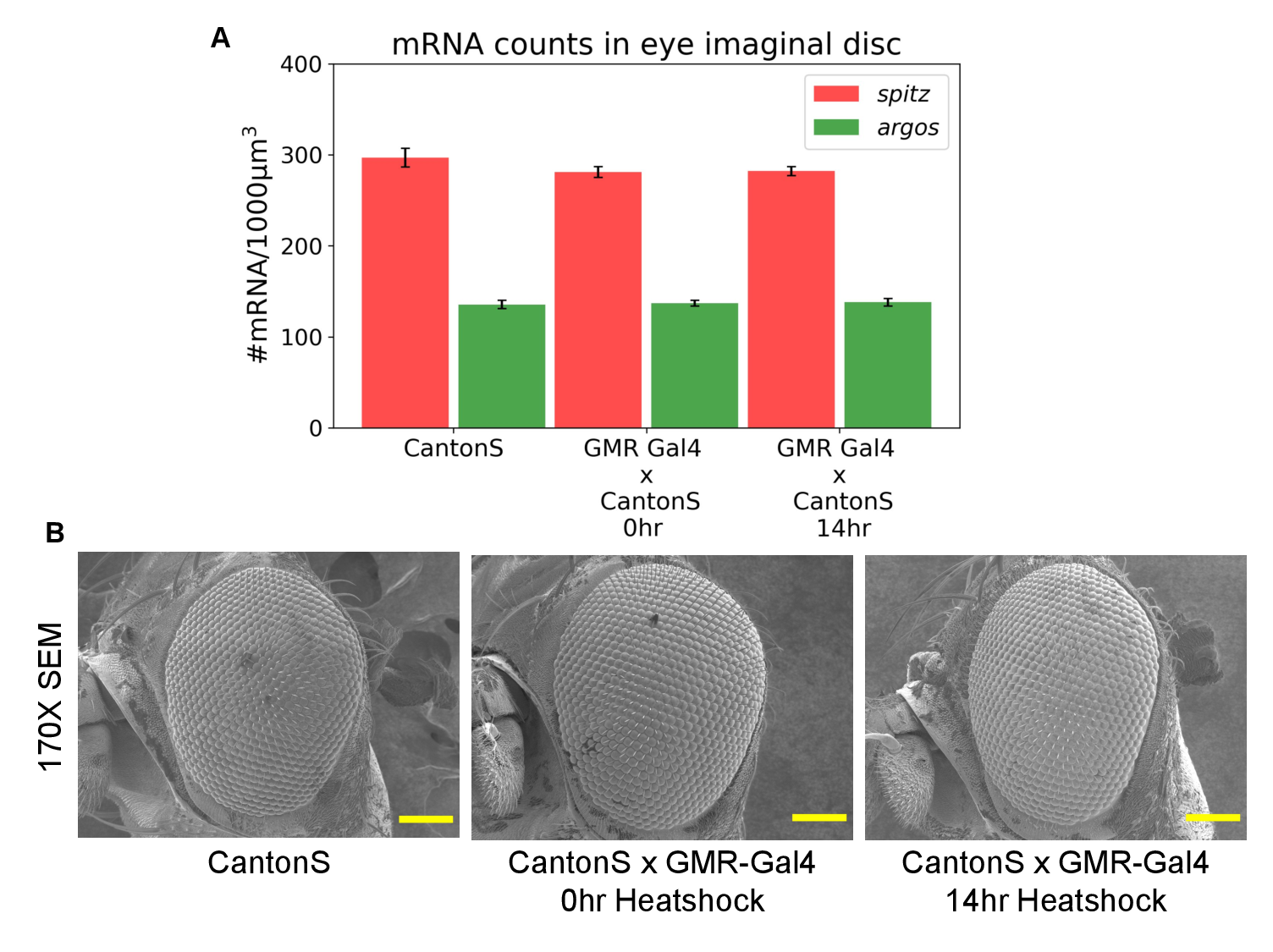

Supplement: S3 Fig — (A) Absolute spitz and argos mRNA numbers are plotted for CantonS and GMR-Gal4 x CantonS flies with and without heatshock at 29°C. The absolute mRNA numbers do not show significant difference (p-values > 0.05 in a Student’s t-test). (N = 8 tissues for all genotypes) Error bars are standard errors of mean. (B) 170X SEM images of the respected genotypes all show perfectly patterned ommatidia. Scale bar is 100μm. The images within the figure panels are created by the authors. Scale bars in Scanning Electron Microscopy images have been provided from knowledge of pixel size and converting appropriately. (TIFF) [file pgen.1010622.s003.tiff]

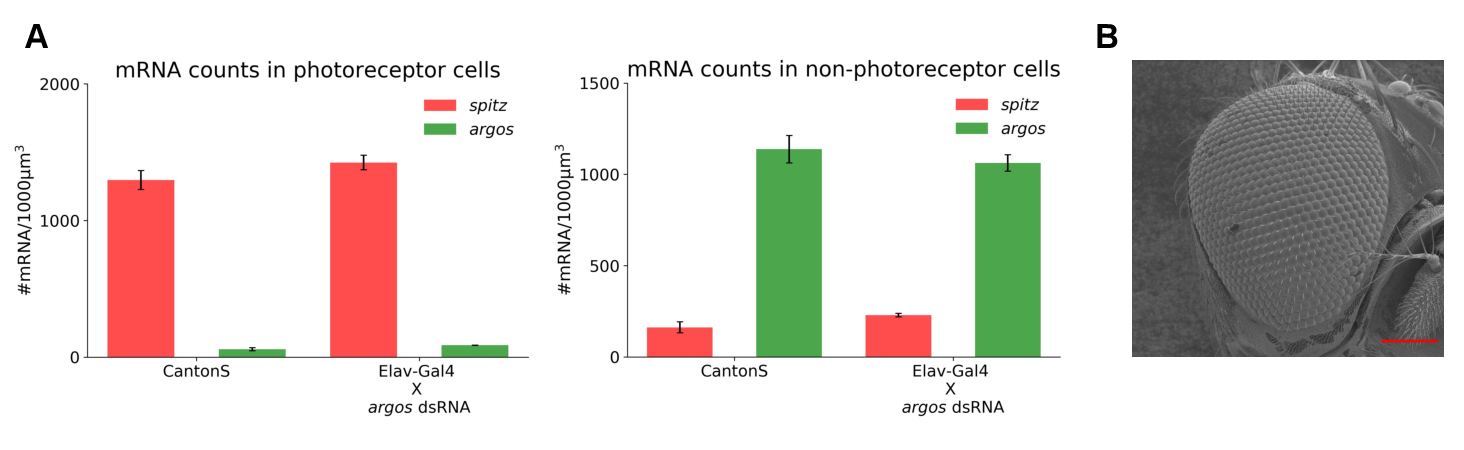

Supplement: S4 Fig — (A) Absolute mRNA counts of spitz and argos in photoreceptors and non-photoreceptors are plotted for CantonS and Elav-Gal4 driving argos dsRNA. There is no significant difference in absolute count of spitz mRNA in photoreceptors and argos mRNA in non-photoreceptors (p-values > 0.05 in a Student’s t-test) (N = 9 tissues) Error bars are standard errors of mean. (B) The adult eye of flies with Elav-Gal4 driving argos dsRNA did not show any defects when compared to the wildtype adult eyes. Scale bar is 100μm. The images within the figure panels are created by the authors. Scale bars in Scanning Electron Microscopy images have been provided from knowledge of pixel size and converting appropriately. (TIFF) [file pgen.1010622.s004.tiff]

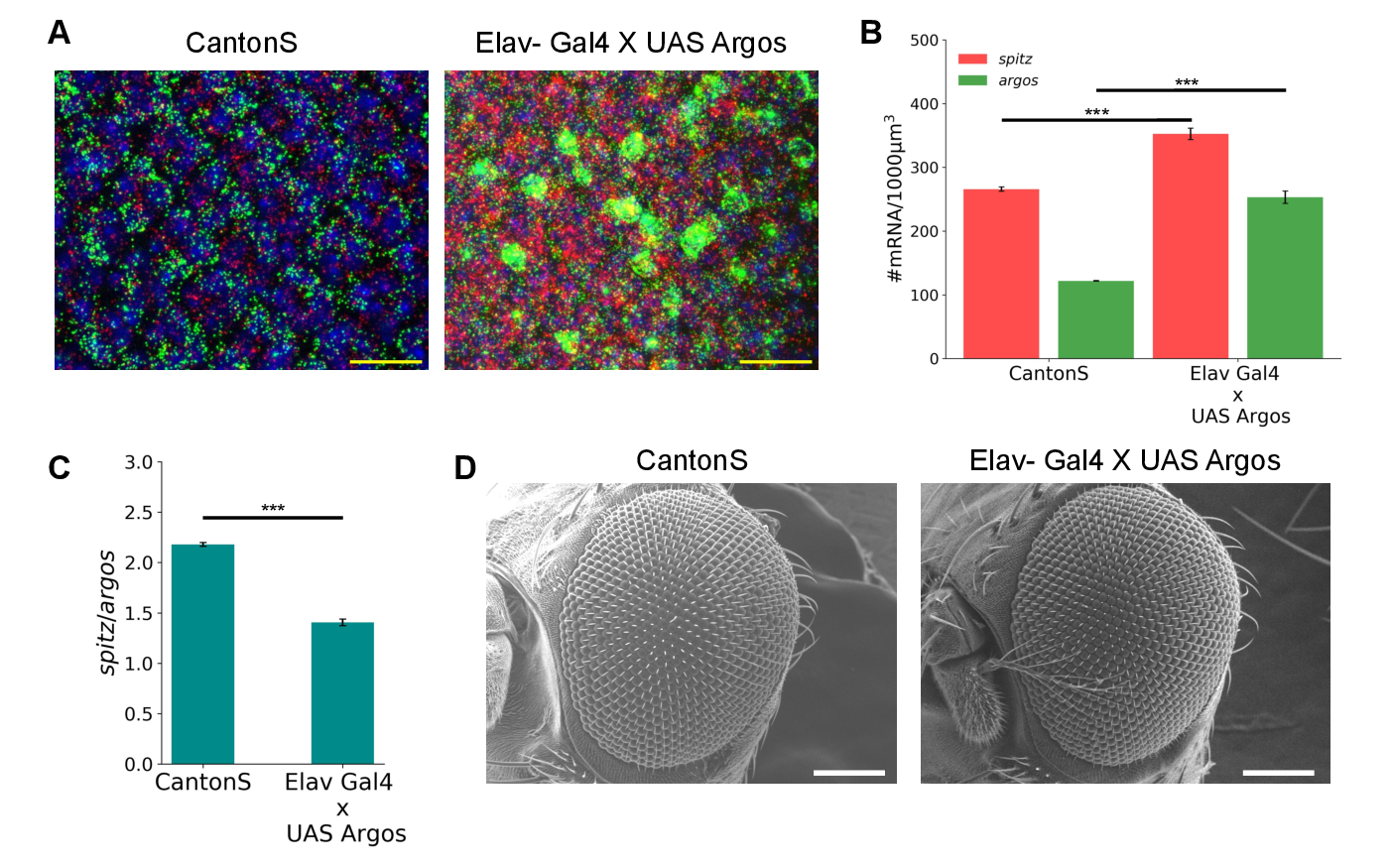

Supplement: S5 Fig — (A) spitz and argos expression pattern in the eye imaginal disc is disrupted by overexpression of UAS Argos by Elav-Gal4 driver in the photoreceptor cells. (B) Absolute spitz and argos mRNA numbers from Elav-Gal4 driving UAS Argos are plotted along with wildtype CantonS. (C) spitz-to-argos ratios from the same eye discs are quantified. N = 8 tissues for all genotypes. Quantification of absolute mRNA numbers and ratios represented in all the above plots were calculated in the eye field irrespective of the cell type (*** indicates p-values < 0.001 in a Student’s t-test). Error bars for all the plots are standard errors of the mean. The change in spitz-to-argos ratio is significantly higher in the Elav-Gal4 driving UAS Argos when compared to wildtype CantonS eye discs (p-values < 0.0001 in a Student’s t-test). (D) 170X SEM images of adult eyes from the wildtype and Elav-Gal4 driving UAS Argos. Scale bar is 10μm in (A) and 100μm in (D). The images within the figure panels are created by the authors. Scale bars in Scanning Electron Microscopy images have been provided from knowledge of pixel size and converting appropriately. Asterisks denoting significance of observed changes have been added to relevant graphs. (TIFF) [file pgen.1010622.s005.tiff]

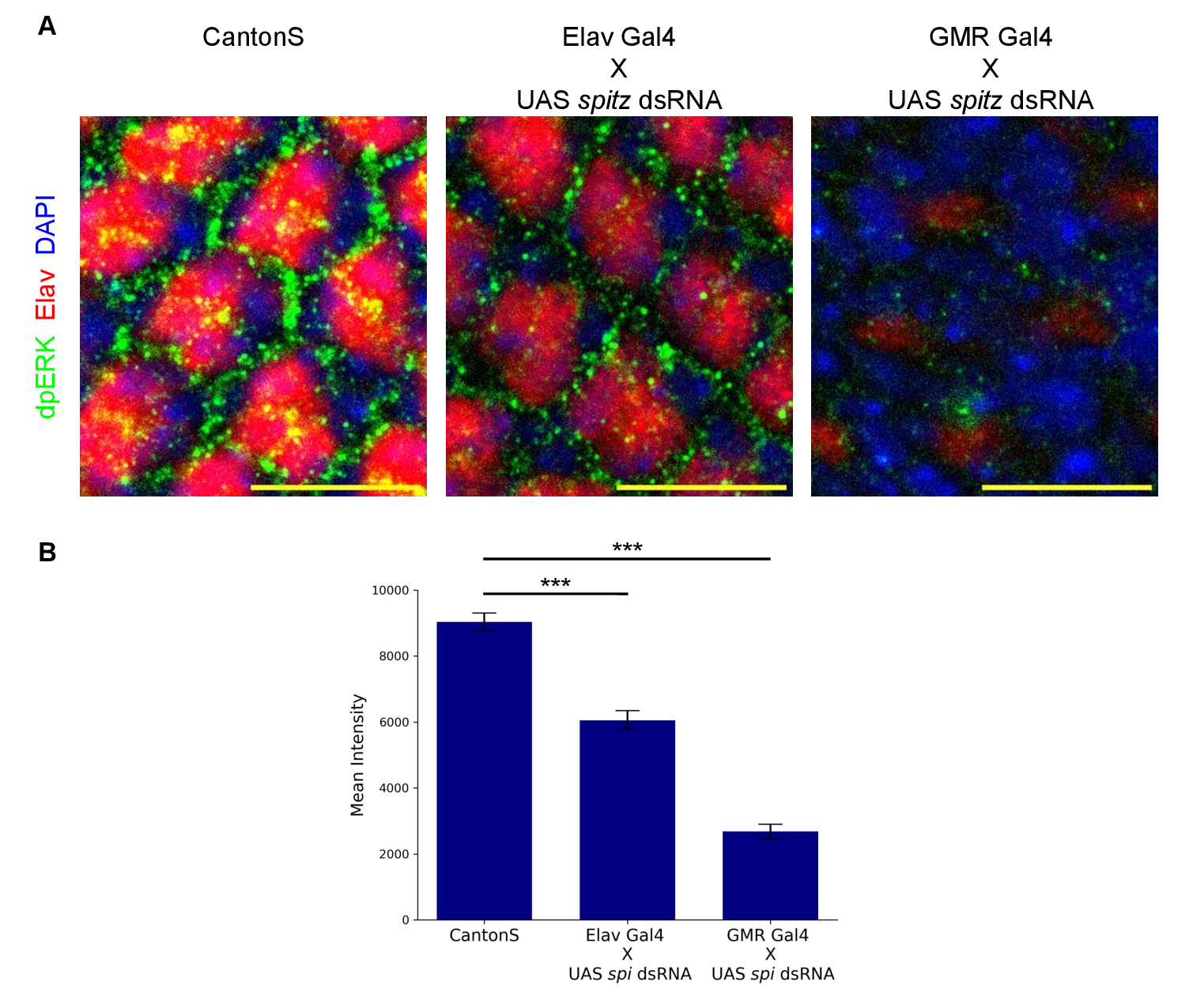

Supplement: S6 Fig — (A) dpERK along with Elav is stained in eye discs from CantonS, Elav-Gal4 driving spitz dsRNA and GMR-Gal4 driving spitz dsRNA. Unlike smFISH which affords absolute mRNA counts, immunofluorescence is a relative measure and it is more difficult to compare across experiments in different strains. But under identical staining and imaging conditions, both the crosses seemed to show significantly reduced dpERK staining compared to CantonS. The reduction was lower for the Elav driver, corresponding to the absence of a phenotype in the adult eye. Residual dpERK signal is observed around the Elav-positive neuronal cells with Elav-Gal4 driving spitz dsRNA. In GMR-Gal4 driving spitz dsRNA, patterned Elav and dpERK staining is lost. (B) Mean Intensities of dpERK in a fixed ROI is plotted here clearly showing the decreased dpERK staining when Elav or GMR-Gal4 drives spitz dsRNA. (*** indicates p-values < 0.001 in a Student’s t-test). N = 6 in all genotypes. Error bars are standard errors of mean. Scale bar is 10μm. The images within the figure panels are created by the authors. Asterisks denoting significance of observed changes have been added to relevant graphs. (TIFF) [file pgen.1010622.s006.tiff]

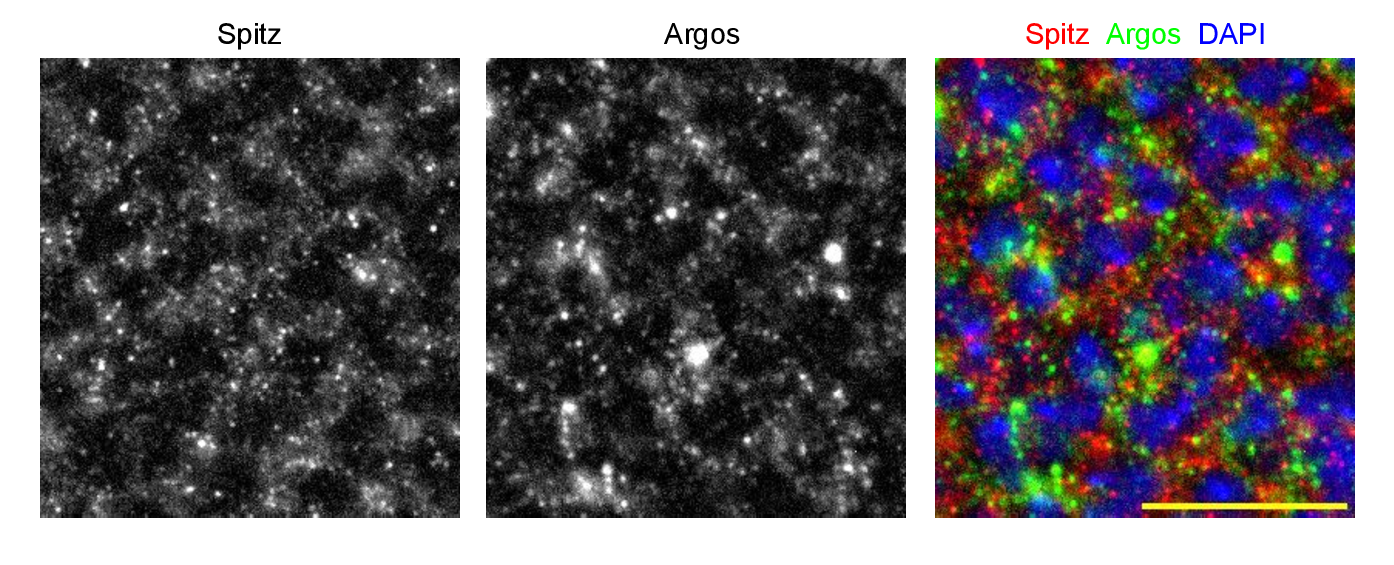

Supplement: S7 Fig — Scale bar is 10μm. Since cleaved Spitz and Argos are diffusible molecules, the staining does not recapitulate the spitz and argos mRNA expression patterns exactly. Moreover, unlike single molecule FISH (smFISH) for RNA that yields absolute mRNA counts, immunofluorescence experiments show only relative changes. The signal intensities captured are dependent on the affinity of the antibodies, quantum yield of the fluorophores used in secondary detection, image acquisition parameters etc. and are different for the two antibodies used. Thus while for a given antibody relative changes can be followed, there is no easy way of comparing staining intensities for antibodies directed against different antigens. Therefore such staining cannot be used for determining expression ratios of Spitz and Argos, unlike with smFISH, which both clearly marks the source cells and also yields absolute transcript counts. To our mind this provides two clear examples of the power of smFISH. The images within the figure panels are created by the authors. (TIFF) [file pgen.1010622.s007.tiff]
