# Supplementary material for: Buffered EGFR signaling regulated by spitz-to-argos expression ratio is a critical factor for patterning the Drosophila eye
Source: PLoS Genet. 2023 Feb 2;19(2):e1010622. doi: 10.1371/journal.pgen.1010622 (PMC9928117; doi:10.1371/journal.pgen.1010622)
Supplement: Attachment — Submitted filename: ReviewerResponse_Pasnuri_et_al_DirectionalEGFRsignaling.pdf [file pgen.1010622.s009.pdf]

<u>Response to reviewers</u>

Reviewer #1: General comments

This manuscript by Nikhita et al reports how EGFR activation is tuned by the relative expression levels of a activation ligand Spitz and a inhibitory regulator Argos to regulate the patterning of the Drosophila eye. By using smFISH to identify the cells that produce the ligands and by a number of genetic means to perturb EGFR signaling, the authors show that the ratio of spitz-to-argos expression is a critical determinant of the final adult eye phenotype, rather than absolute levels of ligands expression. They propose a mechanism in which the relative expression levels of Spitz and Argos determine the extent of EGFR activation, which is critical for periodic ommatidium patterns. The study is potentially interesting, but additional experiments and clarifications are needed to strength the conclusions.

Author response to Reviewer's General Comments

We thank the reviewer for their positive comments and valuable feedback. Revision based on these insightful comments have made the manuscript stronger. We respond to their specific queries below.

Specific question 1

One key observation from the authors is that knocking down spitz in photoreceptor cells by elav-GAL4 does not cause any patterning phenotype, based on which the authors propose that the absolute level of spitz is not important. However, there could be additional EGFR ligands involved in the process, such as keren, which may have redundant functions with spitz. Therefore, the authors appear to make the conclusion based on the assumption that spitz is the only activation ligand involved in the process, which is probably not true.

Should the EGFR signaling activity be affected by either altering spitz or arogos? If spitz is the only ligand, depleting of spitz would cause the loss EGFR signaling.

From the SmFISH results, the authors found that spitz is mainly expressed in PR cells, and argos is exclusively expressed in non-PR cells. However, as the PR cells and non-PR cells are tightly associated with each other, and the cell boundaries are not labeled in the images, it remains possible that spitz and argos are expressed in the same types of cells but with distinct subcellular distributions. Co-staining with a cell membrane marker should help to clarify this issue.

Author response to specific question 1

We thank the reviewer for pointing out the roles of the different EGF ligands in *Drosophila*. The principal EGF Spitz plays a critical role in patterning the eye, and a loss of Spitz from the eye imaginal disc leads to loss of photoreceptors and ommatidia (1). Keren is also known to be expressed in eye imaginal discs and functions in combination with Spitz. Past studies suggest that the relation of Spitz and Keren in the eye disc is more complex than just combinatorial signaling (2). Although it is thought to aid in weak receptor activation and contribute towards a subset of EGFR functions in eye development, Keren null mutants are phenotypically similar to wildtype, which suggests that Keren may not play an essential role

in eye morphogenesis (3). In our experiments, with a skewed stoichiometry between *spitz* and *argos* expression, we do observe rough adult eye phenotypes indicating that they are not rescued by the endogenous expression of Keren.

EGFR signaling activity depends on levels of both Spitz (for receptor activation) and Argos (regulates the availability of free Spitz). Depletion of Spitz would lead to weak EGFR activity since our knockdown experiments significantly decrease but do not entirely abolish *spitz* expression. Reduced Spitz levels may not be sufficient for downstream *argos* expression, which requires high EGFR activity (4). Without Argos sequestering Spitz, the availability of free ligand would be low but might be enough for sustained weak baseline receptor activity.

With the Elav staining and the corresponding patterns of DAPI-stained nuclei, photoreceptor (PR) cells can be clearly distinguished from non-photoreceptor (non-PR) cells. At these high cell packing densities, the nuclei fill up most of the cell. But to conclusively address the reviewer's comment about marking cell boundaries, we stained the eye disc of a wildtype CantonS fly with Elav to mark the neuronal cells in the field along with Dlg (Discs large) (1:1000 dilution, 4F3, DSHB), a membrane associated guanylate kinase to visualize cell boundaries in the tissue field, and DAPI for nuclei (Fig. 1). We observe nuclei overlapping with the pockets of Dlg-stained membrane which are Elav-negative (non-neuronal neighboring cells). These Elav negative nuclei are the non-PR cells that express *argos* mRNA. This figure has now been incorporated in the manuscript as a Supplementary Figure.

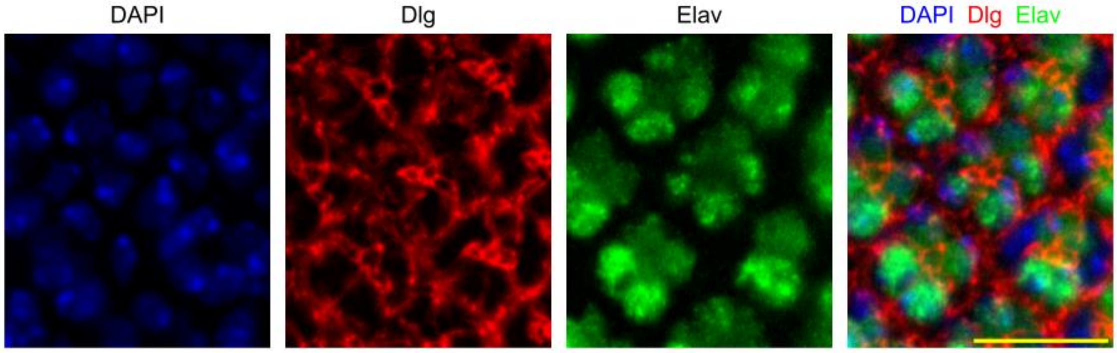

Figure 1: CantonS 3rd instar larval eye disc stained with Dlg and Elav antibody. The nuclei around the clusters of Elav-positive neuronal photoreceptor (PR) cells, are the non-photoreceptor (non-PR) cells. The non-photoreceptors are also separated by Dlg which stains the membrane. The *argos* expression around the clusters of Elav-positive neuronal cells is shown in Figure 1 of the main text. Dlg staining could not be combined with smFISH, but we have previously shown that *spitz* mRNA is primarily found on the Elav-positive PR cell clusters. The scalebar is 5µm.

Specific question 2

Unlike Elav-GAL4, which drives gene expression in PR cells, the authors show that knock-down of spitz by GMR-GAL4, which drives gene expression in both PR and non-PR cells, do cause patterning defects. Should it be indicative that spitz is expressed in non-PR cells and is important for eye patterning?

Author response to specific question 2

The mRNA for *spitz* is expressed in non-PR cells at extremely low levels when compared to the photoreceptor cells (Figure 1C in the manuscript). However, since membrane bound Spitz is cleaved and secreted out of the cell, it can act both cell autonomously and non-autonomously in the developing eye disc and regulates ommatidial patterning, and thus even Spitz from PR cells can exert effects on neighboring non-PR cells. Indeed, in response to Spitz activity, we observe downstream ERK activation and *argos* expression specifically in the non-photoreceptor cells (Supp. Fig. 2A and Fig. 1A and 1B in the manuscript).

Specific question 3

The authors examined the ratio of spitz-to-argos in settings when EGFR signaling is perturbed by expressing EGFR-act or EGFR-DN. But since the effects of these genetic perturbations can no longer be affected by the signaling ligands, the examination of the ligand levels does not seem to be meaningful and informative to support the "buffered signaling" hypothesis.

Author response to specific question 3

We perturbed the *spitz*-to-*argos* ratio using constitutively active and dominant negative forms of EGFR. We should point out that *argos* is a direct target of EGFR signaling and thus these constructs can indeed be used to tune effective available ligand levels. These constructs change the levels of *argos* expression and therefore contribute to corresponding changes in the *spitz*-to-*argos* ratio, allowing the study of buffered EGFR signaling.

Specific question 4

Is the mRNA count in eye imaginal discs equal to that of PR cells plus non-PR cells? Fig. 2B and C show that spitz/argos is about 1, but this is not consistent with the results shown in Fig. 2D and E.

Author response to specific question 4

This difference arises when the ratios are evaluated in specific cell types (PR vs non-PR) or in the full field. Full-field vs cell type-specific counting of mRNA levels has been now explicitly mentioned in the manuscript (lines 180-182).

Specific question 5

Fig. 5: Yan appears to be expressed in Elav+ cells. Does it indicate that EGFR signaling is active in PR cells? But as shown in Fig. 1 and Supplementary Fig. 2, dpERK is restricted to non-PR cells.

Author response to specific question 5

Yan expression as such is not thought of as a proxy for an active EGFR signaling pathway. Rather, the nuclear-to-cytoplasmic relocalization of Yan upon phosphorylation by MAPK is indicative of an active EGFR pathway (5). Since the antibody does not distinguish between phosphorylated and non-phosphorylated forms of Yan, it is difficult to comment on what

fraction of Yan is active. In our experiments it is also hard to distinguish the cytoplasmic and nuclear Yan, but there does seem to be an overall effect on Yan levels. At this point we can say the high Yan cells are surely not as well-patterned as the Elav-positive PR cells. To conclusively address the point brought up by the reviwer, we performed costaining for Elav and Yan in eye discs 3rd instar CantonS larvae. The high Yan cells do not seem to have a clear correspondence to the Elav-positive cells (Fig. 2).

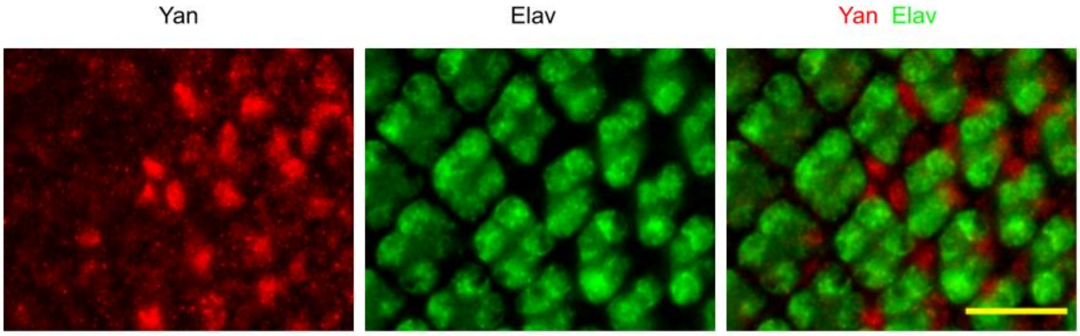

Figure 2: CantonS 3rd instar eye disc stained with Yan and Elav. Elav-positive neuronal cells do not have apparently higher Yan levels in the tissue field. The scalebar is 10 µm.

<u>**Reviewer #2:** General comments</u>

The manuscript presents data to further unravel the role of the Epidermal Growth Factor Receptor, EGFR, for pattern formation in the developing eye of Drosophila. In the eye, the two ligands Spitz and Argos act antagonistically to activate or repress EGFR, respectively. Therefore, expression of the two ligands has to be tightly regulated to ensure correct receptor activation and ultimately correct eye patterning.
Here, the authors used single molecule RNA Fluorescent in situ Hybridisation (smFISH) to quantify spitz and argos transcripts at cellular resolution in the eye discs of third instar larvae of various genetic backgrounds (wildtype, expression of dsRNA and overexpression). Thereby they demonstrate that spitz RNA is restricted to developing photoreceptor cells, while argos RNA is detected in adjacent, non-photoreceptor cells. They further demonstrate that it is not the absolute amount of these RNAs that determines the activation/inhibition of the EGFR pathway, but rather the ratio of the spitz/argos mRNA. Interestingly, the tissue can tolerate a rather wide range of this ratio without showing any defects. This indicates that the system is well buffered to allow proper development even upon small perturbations.
In the second part the authors analyze what they call "the proximal EGFR signalling outcome" to explain the variety of pattern defects achieved upon the different genetic perturbations. This part stays somewhat vague and less clear.

Overall, this manuscript provides novel and interesting results by showing that the ratio of the two ligands Spitz and Argos are important for appropriate EGFR signalling. These data are interesting for scientists interested in EGFR signalling in general. Results presented in the second part are less clear and certainly more interesting for scientists working on pattern formation in the Drosophila eye.

Author response to Reviewer's General Comments

We thank the reviewer for their positive comments and appreciation of the importance of this study. Revision based on their insightful comments have made the manuscript stronger. We respond to their specific queries below.

Specific question 1

Quantification of RNAs: According to Suppl. Fig. 2, they measured the RNA along a line from the morphogenetic furrow to the posterior part of the disc. Along this line, cells/ommatidia are in different stages of development, but they did not take this into account and write (line 142) that there is no "large variation along the anterior to posterior axis". However, I have the impression that in Canton S there is a gradient of argos expression, being higher closer to the morphogenetic furrow.

The difference in developmental stage could be of importance when they overexpress UAS constructs using GMR-Gal4, which then affects cells in different developmental stages. This may also contribute to the variety of eye defects described in the second part of their manuscript.

Author response to specific question 1

As the reviewer points out , *argos* expression does seem slightly higher at the morphogenetic furrow where EGFR activity is also known to be high (6). However, the variability of *argos* expression from morphogenetic furrow to the posterior end is within errorbars as seen in Figure 1C in the manuscript. Though the photoreceptor specification starts just behind the morphogenetic furrow, the PR cells are added into the neuronal cluster sequentially. It could be that the expression level of *argos* stabilizes after a few columns where there are more developed PR clusters. To clarify this point, we plotted the *argos* mRNA counts per 1000um^3 in multiple developmentally staged CantonS eye disc in Fig. 3 here.

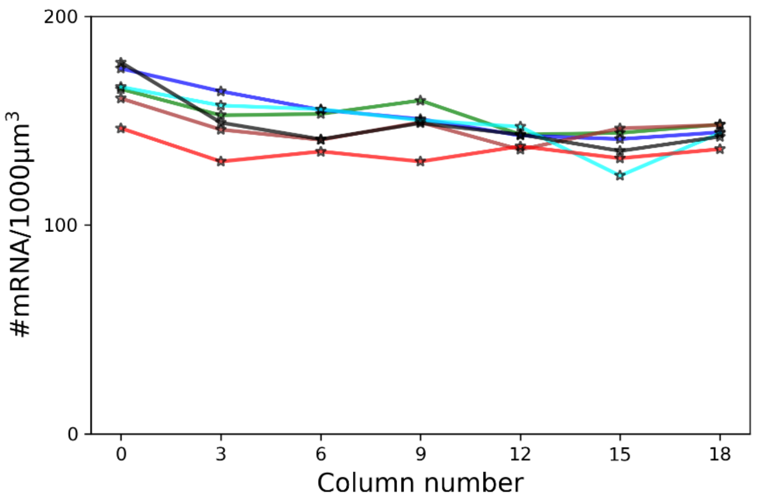

Figure 3: *argos* mRNA from morphogenetic furrow (column 0) to posterior end from CantonS eye discs are plotted. Each line corresponds individual eye disc. Some eye discs have slightly higher *argos* counts near the morphogenetic furrow compared to posterior columns, but not all, making it difficult to talk of a general trend for any given disc; overall, the changes are relatively small.

Specific question 2

Line 24 (and other places, e.g. line 87): what exactly do they mean with "early development"?

Author response to specific question 2

The "early development" in this context means early photoreceptor cell fate determination in the developing 3rd instar larvae. We have incorporated this in the revised manuscript.

Specific question 3

Materials and Methods, line 331-333: How did they stage 3rd instar larvae raised at 18°C? In addition, to inactivate Gal80, they shifted the larvae to 29°C for various times. I guess they prepared the eye discs immediately after the exposure to the higher temperature?

Author response to specific question 3

The crosses were set at 18°C and shifted to new vials every alternate day and maintained at 18°C. Towards the end of the 2nd instar, the larvae were shifted to 29°C for particular time points and immediately shifted back to 18°C and allowed to continue to grow. Even this short period of perturbation in this critical stage could produce large phenotypic defects. Larvae were collected for dissection around mid-to-end 3rd instar. We have included these details in the revised manuscript.

Specific question 4

Line 271: here and in other places the authors use the word "PR cell differentiation". I guess what they mean here (and in other places as well) is PR cell fate specification, since there are more PR cells and less uncommitted cells. I suggest to be more precise in these cases. (In fact they write: "... contributes to higher PR cell differentiation", I guess they mean a higher number of PR cells ... .).

Author response to specific question 4

Thank you for pointing this out. In this manuscript, by PR cell differentiation, we mean PR cell fate specification. This has been changed throughout the revised manuscript.

Specific question 5

Line 178: They write that the ".... spitz to argos ratio ..... was either significantly higher or lower in other genotypes...". How did they determine the significance?

Author response to specific question 5

As mentioned in the methods section, Student's t-test (using GraphPad) provided the significance values which were included in the figure legend. For convenience, we have included asterisks to indicate significance directly on the graphs.

Reviewer #3: General comments

This study focuses on the regulation of EGFR signalling pathway activation in the developing eye imaginal disc. The authors employed a smFISH technique to analyze and compare the transcript expression of, other way difficult to measure, the diffusible ligands of EGFR, spitz and argos. Their results reveal that whereas spitz is expressed specifically in photoreceptors cells, argos expression is detected in the surrounding non differentiated cells. This interesting observation suggests that EGFR signalling is only highly activated on these undifferentiated cells in a non-autonomous manner. In addition, the authors found that the ratio of transcript level of spitz-to-argos is determinant for proper activation of the pathway and for the establishment of the ommatidial pattern. In general, the study provides some interesting observations, especially the non-autonomous effect of spitz and argos, and contributes to a better understanding of eye development and the modulation of the activity of EGFR signaling pathway. However, I have some important concerns regarding the results of this study.

Author response to Reviewer's General Comments

We thank the reviewer for their positive remarks and appreciation of the importance of this study. Revision based on their insightful comments have made the manuscript stronger. We respond to their specific queries below.

Specific question 1

My main concern is that the ratio of spitz/argos regulation of EGFR signalling pathway is mainly based on the fact that depletion of spitz in the PR cells using elavGal4 fails to induce any phenotype in the adult eye. Under these conditions, the levels of spitz are only reduced, rather than abolished, probably due to the strength of either the driver or the dsRNA. Consequently, the activation of argos is proportionally diminished and a mild effect on the determination of PC was observed, as shown in figure 4 where Elav expression and morphogenetic furrow proliferation are slightly affected. In contrast, when spitz was knock down with a more general eye driver, GMR, expression of both spitz and argos are virtually eliminated and EGFR signaling is not activated as Elav expression and morphogenetic furrow proliferation are almost abolished (Fig. 4). In this line, what is the level of dpERK in elav> spitzRNAi compare to GMR>spitzRNAi?

Author response to specific question 1

We thank the reviewer for this comment. As suggested by the reviewer we performed dpERK staining in eye discs of Elav-Gal4 driving *spitz* dsRNA, GMR-Gal4 driving *spitz* dsRNA along with CantonS (Fig. 4). We observed significantly reduced dpERK staining compared to CantonS in both conditions. The reduction was lower for the Elav driver, corresponding to the absence of a phenotype in the adult eye. This figure is now included in the Supplementary Material.

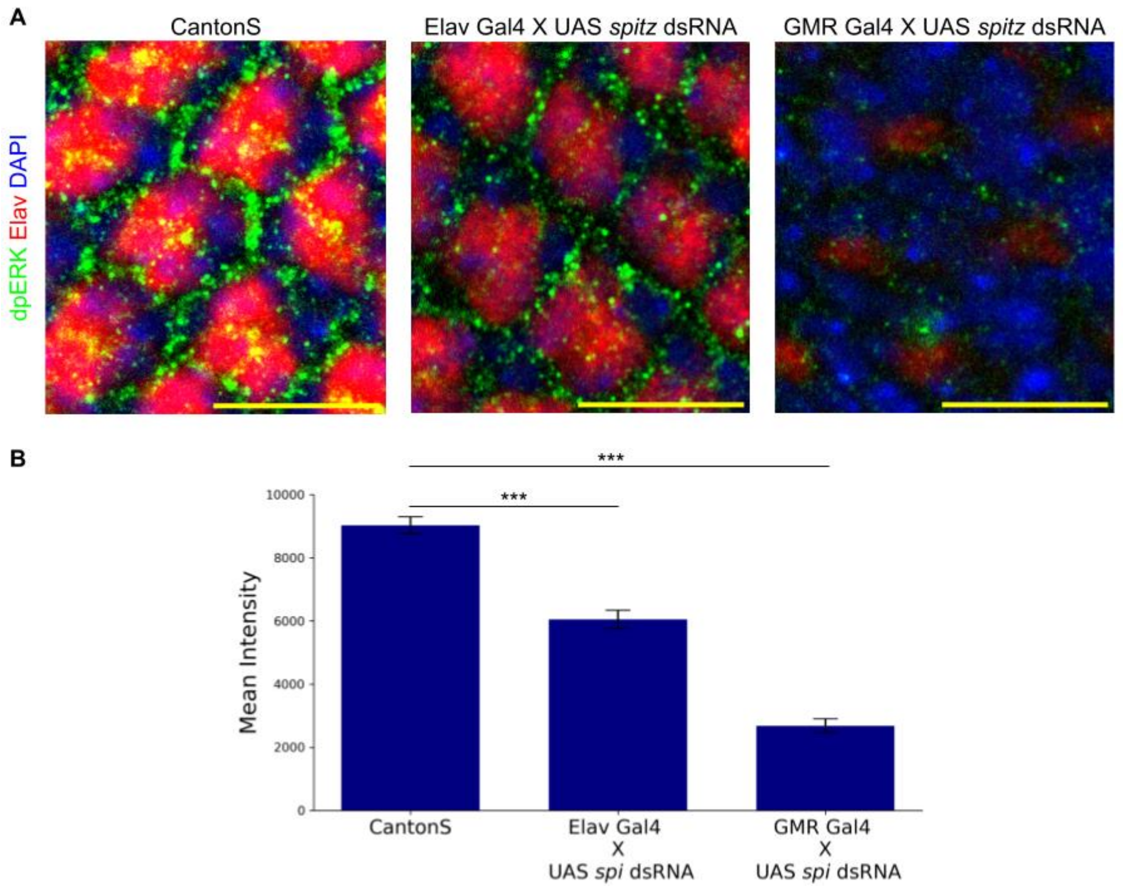

Figure 4: (A) dpERK along with Elav is stained in eye discs from CantonS, Elav Gal4 driving *spitz* dsRNA and GMR Gal4 driving *spitz* dsRNA. Residual dpERK signal is observed around the Elav-positive neuronal cells with Elav Gal4 driving *spitz* dsRNA. In GMR Gal4 driving *spitz* dsRNA, patterned Elav and dpERK staining is lost. (B) Mean Intensities of dpERK in a fixed ROI is plotted here clearly showing the decreased dpERK staining when Elav or GMR Gal4 drives *spitz* dsRNA. (* indicates p-values < 0.001 in a Student's t-test). N=6 in all genotypes. Error bars are standard errors of mean. The scalebar is 10µm.**

We also note that both GMR and Elav Gal4 drivers drive down *spitz* mRNA levels, but neither abrogate expression entirely (see Fig. 2A and 2B in the manuscript). And both in fact cause equally dramatic reduction in *argos* mRNA levels (Figure 2C in the manuscript). *argos* mRNA expression is a proxy for high EGFR activity, and is a more direct readout of EGFR activity than dpERK which can result from general RTK activity. The difference in dpERK levels with GMR and Elav drivers is due to difference in spatial and temporal expression of these drivers, as discussed elsewhere in this document.

Specific question 2

Based on these observations, the authors claim that the phenotype of the eye adult fly is due to the loss of the proper spitz-to-argos expression ratio rather than a mild reduction of EGFR signaling in the first case and almost complete inactivation of the pathway. Since argos expression depends on EGFR signaling activation by spitz, the only factor that is critical to modulate EGFR is the level of spitz. As shown by the authors, reduction of spitz mRNA in PR cells induces a concomitant reduction of argos expression in the NP cells, meaning that spitz:argos ratio only depends on the expression of spitz. Similarly, the authors reach the same conclusion when argos was depleted in the GMR domain. According to the results shown on figure 2, dsRNA of argos seem to work better as

its mRNA transcripts were effectively abolished, as a consequence the ration of spitz/argos is modified and therefore EGFR signaling is wrongly activated. On other words if you eliminate completely argos or spitz is obvious that the ommatidial pattern should be affected and consequently not conclusive.

Author response to specific question 2

We thank the reviewer for this comment. Previous studies show that *spitz* null mutation leads to the loss of ommatidial cells (1). In such a case, there can be no expression of *argos* since its expression is downstream of Spitz activity. If we eliminate *argos* expression, there would be a large amount of free Spitz to bind to the EGF receptor leading to abnormal pathway activation (7). Also, the stoichiometry of *spitz*-to-*argos* is more critical than their absolute expression levels, as also shown in previous studies (8,9). Since we have not eliminated the *spitz* or *argos* expression completely, and tune their expression in a graded manner without eliminating expression completely, we could decipher the buffered expression range for EGFR activation and regulation. (see Figure 3D, 4A and 4D).

Specific question 3

In the same line, the experiments to modified the ratio using EGFR mutants are also not conclusive. Both EGFRCA and EGFDN induced additional effects on the developing eye (both induces less proliferation in the MF and opposite effects on Elav expression, Figure 4). For instance, depletion of spitz and overexpression of EGFRDN produce opposite effects on elav expression. Similarly, overexpression of argos and EGFRDN also produce different eye phenotypes, making difficult to interpret the results in terms of expression ratios. A better approach to investigate the ratio spitz-argos might be to play directly with the ligands level in the cells where they are expressed. Thus, the authors have already shown that, as expected, reducing the levels of spitz in elav positive cells proportionally reduced the expression of argos (Figure 2). However, what happens when spitz is overexpressed? Does the, overexpression of spitz using elavgal4 increase argos expression in NP in the same proportion than in the wt? On the other hand, the authors modified argos levels by overexpressing it in all eye cells. Under these conditions, argos still acts in a non-autonomously manner? How sure the authors are, that the phenotype observed is not produces by and ectopic expression of argos in cells that usually does not produce this protein rather than a change in the expression ratio? Is it possible to increase argos levels specifically in NPR cells? What is the effect of overexpressed of argos in PR cells, using elavGal4?
Finally, I do not understand why the authors claim that the ratio spitz/argos is around 2,1. If you compared the expression of spitz in PR cells vs. argos in the NP cells the mRNA/1000nm3 are nearly the same. Therefore, the ratio is around 1:1 as previously reported.

Author response to specific question 3

We appreciate this point brought up by the reviewer. As of now we do not have access to a line that overexpresses *spitz* or a Gal4 driver specific to non-PR cells. But to address the point raised by the reviewer and ectopically express *argos* in the photoreceptor cells, we drove the expression of UAS-Argos using the Elav-Gal4 driver. We observe the expression of *argos* in the photoreceptor cells through smFISH (Fig. 5A). Quantification of absolute *spitz* and *argos*

mRNA counts (Fig. 5B) and *spitz*-to-*argos* expression ratio (Fig. 5C) showed that progeny from Elav-Gal4 driving UAS Argos have lower expression ratio (1.40) compared to wildtype eye discs (p-values lower than 0.0001 in a Student's t-test). Though the expression ratio decreased, there was no phenotypic change in the adult flies (Fig. 5D). This could be because the ratios did not fall below the lower limit of the *spitz*-to-*argos* buffer range (1 to 3, with a mean of 2). Phenotypic changes are observed only when the ratio falls below 1 or goes above 3 (Figures 3F, 4C and 4F in the manuscript). This is remarkable, because clearly the UAS-Argos cassette is working as evidenced by both the images and the RNA counts. The *spitz* mRNA levels are somewhat higher in this cross. This feedback to *spitz* mRNA levels was somewhat unexpected. This observation could further emphasize the importance of the *spitz*-to-*argos* ratio in determining the strength of the EGFR pathway. This figure is now included as Supplementary Figure 6 in the manuscript.

Finally, while Argos binds to secreted Spitz in a 1:1 stoichiometry (7), this may not indicate that Argos and Spitz are expressed in a 1:1 ratio. In our case in a given tissue volume (without distinguishing cell types), the absolute *spitz* mRNA expression is approximately twice that of *argos* mRNA.

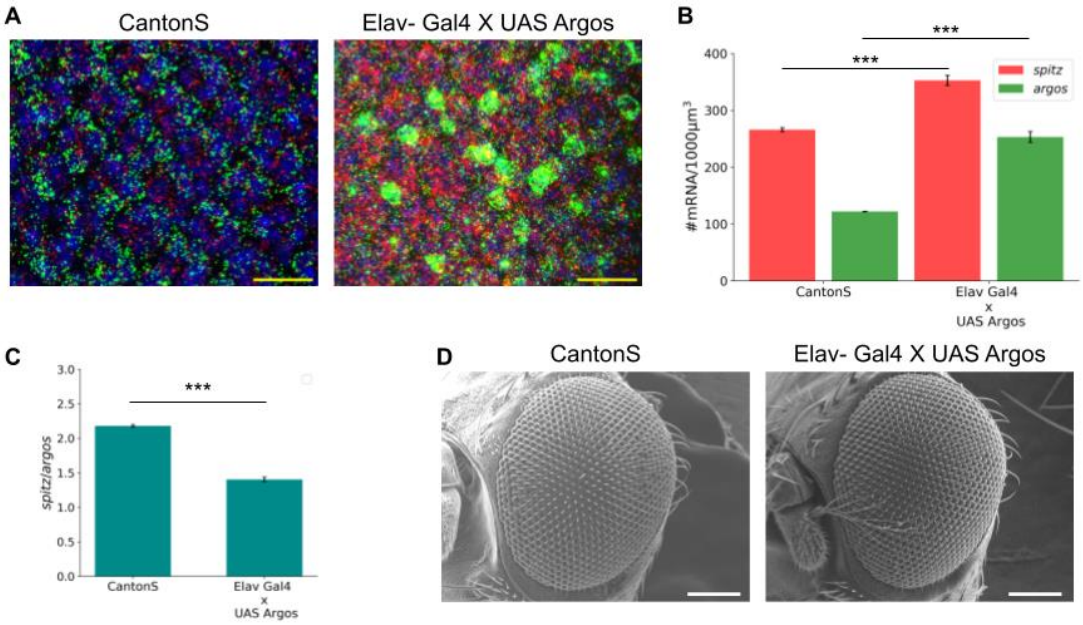

Figure 5: (A) *spitz* and *argos* expression pattern in the eye imaginal disc is disrupted by overexpression of UAS Argos by Elav-Gal4 driver in the photoreceptor cells. (B) Absolute *spitz* and *argos* mRNA numbers from different crosses are plotted. (C) *spitz*-to-*argos* ratios from the same eye discs are quantified. N=8 tissues for all crosses. Quantification of absolute mRNA numbers and ratios represented in all the above plots were calculated in the eye field irrespective of the cell type. N=6 for all genotypes. Error bars for all the plots are standard errors of the mean. The change in *spitz*-to-*argos* ratio is significantly higher in the Elav-Gal4 driving UAS Argos when compared to wildtype CantonS eye discs (* indicates p-values < 0.001 in a Student's t-test). (D) 170X SEM images of adult eyes from the wildtype and Elav-Gal4 driving UAS Argos. The scalebar is 10µm in (A) and 100µm in (D).**

Specific question 4

Addition of the expression domains of ElavGal4 and GMRgal4 might help to non-specialist readers to better understand the logic of the experiments.

Author response to specific question 4

GMR is expressed in all cells posterior to the morphogenetic furrow (10) and any expression cassette under the UAS is expressed strongly. Whereas, Elav is expressed only in neuronal cells and the expression is strongest towards the posterior end of the eye disc (11). This has now been explicitly indicated in the text.

Specific question 5

In Figure 1 the authors show the complementary expression of spitz and argos in Elav positive cells and non-expressing Elav cells. However, whereas the non-overlapping expression of the two ligands is quite clear, the fact that are elav positive cells or not is not properly shown as in the merge panel is difficult to distinguish the blue color.

Author response to specific question 5

A zoomed-in image of *spitz* and *argos* mRNA is provided in the supplementary text (Supp. Fig. 1B). The current image has been modified so that the Elav expression is clear.

Specific question 6

In figure 2, depletion of argos under the control of GMRGal4 induce a significance increase in the expression of spitz. However, overexpression of neither EGFRDN nor EGFRCA do not change the expression of spitz, suggesting a different effect on signalling when using these constructs.

Author response to specific question 6

Depletion of *argos* using GMR Gal4 induced the *spitz* expression in the eye disc unlike $EGFR^{CA}$ or $EGFR^{DN}$. While *spitz* expression itself is not a target of EGFR activity (4), indeed, this could indicate a compensatory signaling mechanism acting to buffer the perturbation caused in the developing eye disc. Figure 5 in this response (see above), where we used Elav Gal4 to drive UAS Argos, also shows an increase in *spitz* expression.

Specific question 7

Significance of the differences in mRNA counts should be added in the graphs of all figures.

Author response to specific question 7

Following the reviewer's suggestion, we have now added asterisks denoting significance in all the graphs in the revised manuscript.

Specific question 8

Since there are argos and spitz fusion proteins or Ab. Could and extracellular staining of the proteins use as a tool to determine the ratio spitz/argos?

Author response to specific question 8

Following the reviewer's suggestion, we procured Spitz (extracellular domain specific) and Argos antibodies and stained 3rd instar eye discs. Staining of Argos using antibody does not show clear expression pattern similar to the expression patterns of *argos* mRNA (Fig. 6). However, the depositor note for the Argos antibody says that the staining is not convincing (https://dshb.biology.uiowa.edu/Anti-Argos-85-2-16). On the other hand, Spitz staining did not show specific enrichment in the photoreceptor cells (Fig. 6), but this could be because uncleaved Spitz is present on the membrane of PR cells. This is further complicated by the fact that cleaved Spitz and Argos are diffusible molecules. Moreover, unlike single molecule FISH (smFISH) for RNA that yields absolute mRNA counts, immunofluorescence experiments show only relative changes. The signal intensities captured are dependent on the affinity of the antibodies, quantum yield of the fluorophores used in secondary detection, image acquisition parameters etc. and are different for the two antibodies used. Thus while for a given antibody relative changes can be followed, there is no easy way of comparing staining intensities for antibodies directed against different antigens. Therefore, unlike with smFISH, antibody staining is not suitable for determining expression ratios of Spitz and Argos, , To our mind this provides two clear examples of the power of smFISH. We thank the reviewer for suggesting this experiment. This new experiment has now been included in the Supplementary Material.

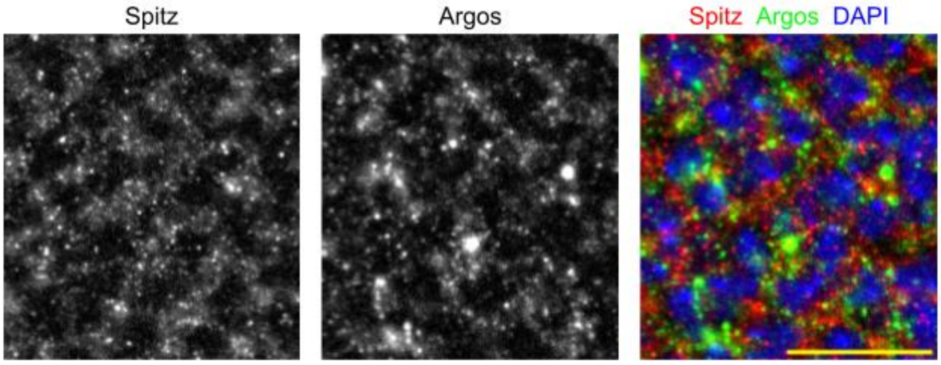

Figure 6: CantonS 3rd instar eye discs are stained with Spitz (Rat anti-Spitz, DSHB) and Argos (Mouse anti-Argos 85/2/16, DSHB) antibodies. Since cleaved Spitz and Argos are diffusible molecules, the antibody staining did not capture their cell-type specific gene expression patterns. Spitz and Argos antibodies were used at 1:500 dilution and Goat anti-Rat 488 [Invitrogen, A11006] and Goat anti-Mouse 594 [Invitrogen, A11032] were used at 1:1000 dilution. The scalebar is 10µm.

Specific question 9

The changes in the expression of spitz and argos is shown in the overexpression of EGFRCA but not in the UASargos or UASEGFRDN.

Author response to specific question 9

Absolute *spitz* and *argos* numbers have been provided for UAS Argos (Fig. 4A in the manuscript) and UAS EGFR^{DN} (Fig. 4D in the manuscript).

Specific question 10

Adult heads are not properly orientated anterior-posterior in Figure 3F, 4F and SFig 2B.

Author response to specific question 10

We thank the reviewer for pointing this out. We have now aligned the images in the revised manuscript.

References

1. Freeman M. The spitz gene is required for photoreceptor determination in the Drosophila eye where it interacts with the EGF receptor. Mech Dev. 1994;48(1):25–33.
2. Brown KE, Freeman M. Egfr signalling defines a protective function for ommatidial orientation in the Drosophila eye. Development. 2003;130(22):5401–12.
3. Brown KE, Kerr M, Freeman M. The EGFR ligands Spitz and Keren act cooperatively in the Drosophila eye. Dev Biol. 2007;307(1):105–13.
4. Wasserman JD, Freeman M. An autoregulatory cascade of EGF receptor signaling patterns the Drosophila egg. Cell. 1998;95(3):355–64.
5. Rebay I, Rubin GM. Yan functions as a general inhibitor of differentiation and is negatively regulated by activation of the Ras1/MAPK pathway. Cell. 1995;81(6):857–66.
6. Gabay L, Seger R, Shilo BZ. In situ activation pattern of Drosophila EGF receptor pathway during development. Science (80-). 1997;277(5329):1103–6.
7. Klein DE, Nappi VM, Reeves GT, Shvartsman SY, Lemmon MA. Argos inhibits epidermal growth factor receptor signaling by ligand sequestration. Nature [Internet]. 2004;430(7003):1040–4. Available from: http://dx.doi.org/10.1038/nature02840
8. Schweitzer R, Howes R, Smith R, Shilo B-Z, Freeman M. Inhibition of Drosophila EGF receptor activation by the secreted protein Argos. Nature. 1995;376(August):699–702.
9. Taguchi A, Sawamoto K, Okano H. Mutations modulating the Argos-regulated signaling pathway in Drosophila eye development. Genetics. 2000;154(4):1639–48.
10. Li WZ, Li SL, Zheng HY, Zhang SP, Xue L. A broad expression profile of the GMR-GAL4 driver in Drosophila melanogaster. Genet Mol Res. 2012;11(3):1997–2002.
11. Yao KM, White K. Neural specificity of elav expression: Defining a Drosophila promoter for directing expression to the nervous system. J Neurochem. 1994;63(1):41–51.
12. Domínguez M, Wasserman JD, Freeman M. Multiple functions of the EGF receptor in Drosophila eye development. Curr Biol [Internet]. 1998;8(19):1039–48. Available from: http://www.ncbi.nlm.nih.gov/pubmed/9768358
